# Assessing Tumor Size by MRI and Pathology in Type I Endometrial Carcinoma to Predict Lymph Node Metastasis

**DOI:** 10.7759/cureus.23135

**Published:** 2022-03-14

**Authors:** Maria Ali, Mehwish Mumtaz, Zehra Naqvi, Rabia Farooqui, Sania A Shah

**Affiliations:** 1 Obstetrics and Gynaecology, Liaquat National Hospital and Medical College, Karachi, PAK; 2 Paediatrics, Abbasi Shaheed Hospital, Karachi, PAK

**Keywords:** type i endometrial carcinoma, lymphadenectomy, lymph node metastasis, tumor size, endometrial carcinoma

## Abstract

Introduction

Lymphatic spread is the most common route of spread of endometrial carcinoma, and the most frequently involved lymph nodes are those of the external iliac group. MRI is one of the best imaging tools for the preoperative evaluation of patients with endometrial carcinoma. The objective of the current study is to analyze the relationship between tumor size and lymph node metastasis in patients with type I endometrial carcinoma.

Methods

This is a prospective observational study performed in the Department of Obstetrics and Gynaecology at Liaquat National Hospital, Karachi, Pakistan. The duration of the study was from January 2020 to January 2021. During this period, 86 patients with biopsy-proven type I endometrial carcinoma were selected. Tumor size was measured by MRI. All participants underwent a total abdominal hysterectomy, bilateral salpingo-oophorectomy, and bilateral pelvic lymphadenectomy. Histopathological evaluation was performed according to the College of American Pathologists (CAP) protocols, and staging was performed using the 2009 International Federation of Gynecology and Obstetrics (FIGO) staging system. Lymph nodes were considered positive or negative, irrespective of their number.

Result

Of the 86 patients, 25 (29.1%) had positive lymph node metastasis. The mean tumor size with positive lymph node metastasis by MRI and histopathology was 7.86 cm and 10.21 cm, respectively. Tumor size determined by MRI and histopathology was significantly associated with lymph node metastasis (p < 0.01 and p < 0.01, respectively). Tumor size was positively correlated with lymph node metastasis (r = 0.715). The cutoff value of >6.5 cm by MRI was established as the statistically significant differentiator of lymph node metastasis. The calculated sensitivity and specificity were 88% and 90.16%, respectively, with an area under the curve (AUC) of 0.920. The cutoff value of >8 cm by histopathology was established as the statistically significant differentiator of lymph node metastasis. The calculated sensitivity and specificity were 80% and 88.52%, respectively, with an AUC of 0.907.

Conclusion

Our results showed that lymph node metastasis in patients with type I endometrial carcinoma can be predicted by tumor size. This may help incorporate adequate surgical skills and management plans in the treatment course of type I endometrial carcinoma.

## Introduction

Endometrial cancer is the sixth most common cancer worldwide. In developed countries, this cancer is ranked as the fourth most common cancer among females. Over the last three decades, the incidence of endometrial cancer has increased, and women aged 50 years and older account for 90% of all cases, with a median age of 63 years [1]. In Pakistan, endometrial cancer is considered the third most common malignancy in females, after carcinoma of the cervix and ovary [2]. The five-year and 10-year survival rates are 82% and 79%, respectively [3]. When detected at an early stage, patients with endometrial adenocarcinoma have a survival rate of 90% in developed countries [4]. Although the survival rate has improved, in the last decade, mortality from endometrial cancer has reached 20% [5].

Multiple risk factors, such as hypertension, early menarche, late menopause, nulliparity, infertility, and unopposed estrogen exposure, are linked with endometrial cancer. Tamoxifen, a selective estrogen receptor modulator, may cause endometrial polyps and hyperplasia, which leads to endometrial carcinoma in postmenopausal women. A higher body mass index (BMI) is also an important risk factor. It has been suggested that 60% of endometrial carcinoma cases in Europe are due to increased weight gain [5,6]. Women with polycystic ovarian syndrome (PCOS) have a fourfold increased risk of developing endometrial carcinoma compared with women without PCOS [7]. Lynch syndrome is also related to endometrial carcinoma with an estimated 60% overall lifetime risk [8].

The risk factors for endometrial cancer relapse include both uterine and extrauterine factors. Uterine risk factors include tumor histology, tumor size, lymphovascular invasion, cervical stroma invasion, and invasion depth. Extrauterine risk factors include peritoneal cytology, nodal involvement (pelvic and para-aortic), intraperitoneal metastasis, and ovarian metastasis [9-12]. However, volume index, serum CA-125 levels, myometrial invasion, and grade are the most significant risk factors for lymph node metastasis [13].

Two different types of endometrial carcinoma have been defined based on molecular profile, clinical course, and histopathology. Type I is the most common (70%-80%) type in obese women and is endometrioid, moderately to well-differentiated, diploid, hormone receptor-positive, low-grade, and localized, and has a favorable prognosis. Type II (20%-30%) occurs in nonobese women and is non-endometrioid, aneuploid, hormone receptor-negative, high-grade, and poorly differentiated, and has a high metastatic risk and poor prognosis [6,14]. According to recent guidelines, endometrial cancer is divided into high-risk/undifferentiated cancer (for this, complete surgical staging is required) and low-risk/endometrioid type, which is an initially infiltrating cancer (requires a less invasive approach) [1]. A significant association may be observed between tumor size and lymph node metastasis [15].

Lymphatic spread is the most common route. The lymph nodes involved are the pelvic, obturator, inguinal, presacral, external, internal, common iliac, and para-aortic lymph nodes, i.e., paracaval, precaval, retrocaval, and right-lateral aortic nodes. The most commonly involved lymph nodes are those of the external iliac group [4].

The most appropriate imaging tool for the preoperative evaluation of patients with endometrial carcinoma is MRI, as this modality provides insight into local staging, cervical stromal invasion, myometrial invasion, and adnexal metastasis, along with evidence of vaginal, bladder, and rectal involvement [16].

The objective of this study is to analyze the relationship between tumor size and lymph node metastasis in patients with type I endometrial carcinoma.

## Materials and methods

Study design, settings, and duration of the study

This is a prospective observational study that was performed in the Department of Obstetrics and Gynaecology at Liaquat National Hospital, Karachi, Pakistan, from January 1, 2020, to January 1, 2021.

Inclusion criteria

All primary patients with biopsy-proven type I endometrial carcinoma were selected. The disease was stage I according to MRI-based staging.

Exclusion criteria

Patients with type II endometrial carcinoma, those with stage II and above by MRI, those with concomitant ovarian pathology, and patients who had received preoperative chemotherapy and radiotherapy were excluded.

Data collection procedure

Approval was granted from the hospital’s ethical review committee (approval number #0469-2019-LNH-ERC). All recruited patients provided informed consent. Tumor size was measured by MRI as the single largest dimension of the tumor by a single expert radiologist at the radiology department of Liaquat National Hospital.

In all, 86 patients with biopsy-proven type I endometrial carcinoma were selected. All participants underwent a total abdominal hysterectomy, bilateral salpingo-oophorectomy, and bilateral pelvic lymphadenectomy.

The samples were then sent to the histopathology department where a detailed histopathological evaluation was performed according to protocols of the College of American Pathologists (CAP) (version 4.1.0.2, February 2020) for the assessment of specimens derived from patients with endometrial carcinoma [17]. Patients were staged according to the 2009 International Federation of Gynecology and Obstetrics (FIGO) staging criteria [18]. Lymph nodes were considered positive or negative, irrespective of their number.

Data analysis procedure

After collection, data were analyzed using Statistical Package for the Social Sciences version 25 (IBM Corp., Armonk, NY, USA). The distribution of continuous data was compared using an independent t-test. The frequencies of categorical measures among different groups were compared using Fisher’s exact test, while the Chi-square test was applied for qualitative variables. Statistical significance was determined by a p-value < 0.05. The correlation between tumor size and lymph node metastasis was determined using the Pearson correlation coefficient. The diagnostic criterion for lymph node metastasis was established using the Youden index.

## Results

Patient characteristics

This study enrolled 86 patients who were diagnosed with type I endometrial carcinoma. The mean patient age was 59.08 ± 9.38 years, and the mean BMI was 23.46 ± 3.31 kg/m^2^; seven selected patients were diabetic (8.1%), 14 (16.3%) were hypertensive, 77 were menopausal (89.5%), and seven (8.1%) had postmenopausal bleeding (Table 1).

**Table 1:** Descriptive statistics of the study population SD: standard deviation

Patient characteristics	Values
Age (years)	
Mean ± SD	59.08 ± 9.38
Groups	
<45 years, n (%)	7 (8.1)
>45 years, n (%)	79 (91.9)
Height (cm), mean ± SD	160.96 ± 12.94
Weight (kg), mean ± SD	61.15 ± 8.05
BMI (kg/m^2^), mean ± SD	23.46 ± 3.31
Normal, n (%)	40 (46.5)
Overweight, n (%)	23 (26.7)
Obese, n (%)	23 (26.7)
Diabetes	
Yes, n (%)	7 (8.1)
Hypertension	
Yes, n (%)	14 (16.3)
Menopausal status	
Menopausal, n (%)	77 (89.5)
Menopausal bleeding	
Yes, n (%)	7 (8.1)

Tumor characteristics

The median (interquartile range (IQR)) tumor size by MRI was 5 (3.53) cm and ranged from 2.50 cm to 10.50 cm. The median (IQR) tumor size by histopathology was 6.25 (4.43) and ranged from 2.20 cm to 15 cm. Thirty-four patients had grade I tumors (36%), and 52 patients had grade II tumors (52%). Fifty-five (64%) patients had stage IB disease, and 25 patients (29.1%) showed lymph node metastasis (Table 2).

**Table 2:** Descriptive statistics of tumors SD: standard deviation, IQR: interquartile range

Tumor characteristics	Values
Tumor size by MRI (cm)	
Mean ± SD	5.51 ± 2.25
Median (IQR)	5 (3.53)
Min–max	2.50–10.50
Tumor size by histopathology (cm)	
Mean ± SD	6.97 ± 3.09
Median (IQR)	6.25 (4.43)
Min–max	2.20–15
Tumor grade	
Grade I, n (%)	34 (39.5)
Grade II, n (%)	52 (60.4)
Tumor stage	
IA, n (%)	31 (36)
IB, n (%)	55 (64)
Lymphatic metastasis	
Positive, n (%)	25 (29.1)
Negative, n (%)	61 (70.9)

Comparison of lymphatic metastasis with different variables

A statistically significant association was found between tumor size (by MRI and histopathology) and lymph node metastasis (p < 0.01 and p < 0.01, respectively). No statistically significant association was found between lymph node metastasis and comorbidities such as hypertension (p = 0.537) and diabetes (p = 0.101) (Table 3).

**Table 3:** Comparison of lymphatic metastasis with different variables *Independent t-test was applied. **Chi-square test was applied. ***Fisher’s exact test was applied.

Variables	Lymphatic metastasis		
	Positive	Negative	p-value
Age (years), mean ± SD*	61.16 ± 8.82	58.22 ± 9.54	0.190
Groups***			
≤45 years, n (%)	1 (4)	6 (9.8)	0.668
>45 years, n (%)	24 (96)	55 (90.2)	
BMI (kg/m^2^), mean ± SD*	23.15 ± 2.62	23.59 ± 3.56	0.578
Groups**			
Normal, n (%)	12 (48)	28 (45.9)	0.613
Overweight, n (%)	8 (32)	15 (24.6)	
Obese, n (%)	5 (20)	18 (29.5)	
Tumor size by MRI (cm), mean ± SD*	7.86 ± 1.46	4.55 ± 1.75	<0.01
Tumor size by histopathology (cm), mean ± SD*	10.21 ± 2.54	5.65 ± 2.19	<0.01
Diabetes***			
Yes, n (%)	0 (0)	7 (11.5)	0.101
No, n (%)	25 (100)	54 (88.5)	
Hypertension***			
Yes, n (%)	5 (20)	9 (14.8)	0.537
No, n (%)	20 (80)	52 (85.2)	
Menopausal status***			
Menopausal, n (%)	23 (92)	56 (91.8)	1.000
Menopausal bleeding***, n (%)	2 (8)	5 (8.2)	
Tumor grade**			
Grade I, n (%)	1 (4)	33 (54.1)	<0.01
Grade II, n (%)	24 (96)	28 (45.9)	
Tumor stage**			
IA, n (%)	0 (0)	31 (50.8)	<0.01
IB, n (%)	25 (100)	30 (49.2)	

Comparison of tumor size and lymph node metastasis

**Figure 1:**
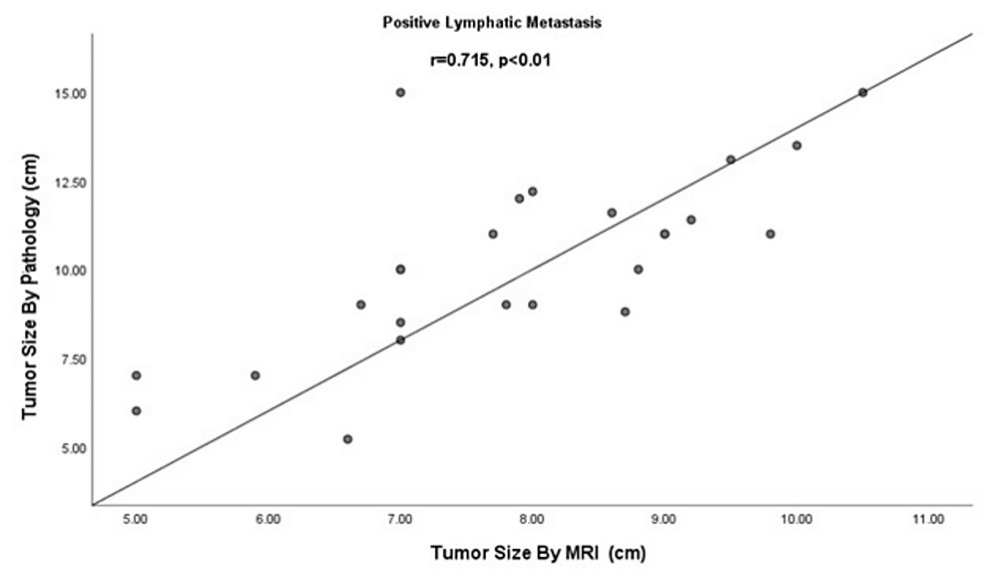
Comparison of tumor size and lymph node metastasis r = Pearson correlation coefficient

Results when analyzed showed accordance between tumor size assessed by MRI and histopathology in comparison to lymph node metastasis as shown in Figure 1. Lymph node metastasis increases with increasing tumor size. A correlation was determined by the Pearson correlation coefficient.

**Figure 2:**
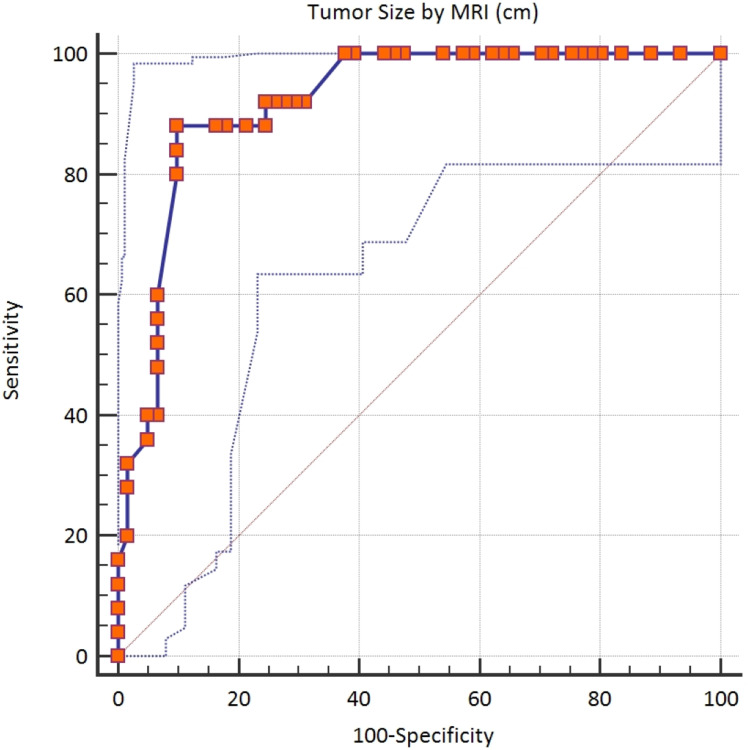
Youden index

According to the Youden index, the cutoff value of >6.5 cm by MRI was established as a statistically significant differentiator of lymph node metastasis. The sensitivity and specificity were calculated to be 88% and 90.16%, respectively, with an area under the curve (AUC) of 0.920 (Table 4 and Figure 2).

**Table 4:** Youden index

Youden index	0.7816
Associated criterion	>6.5
Sensitivity	88%
Specificity	90.16%

**Figure 3:**
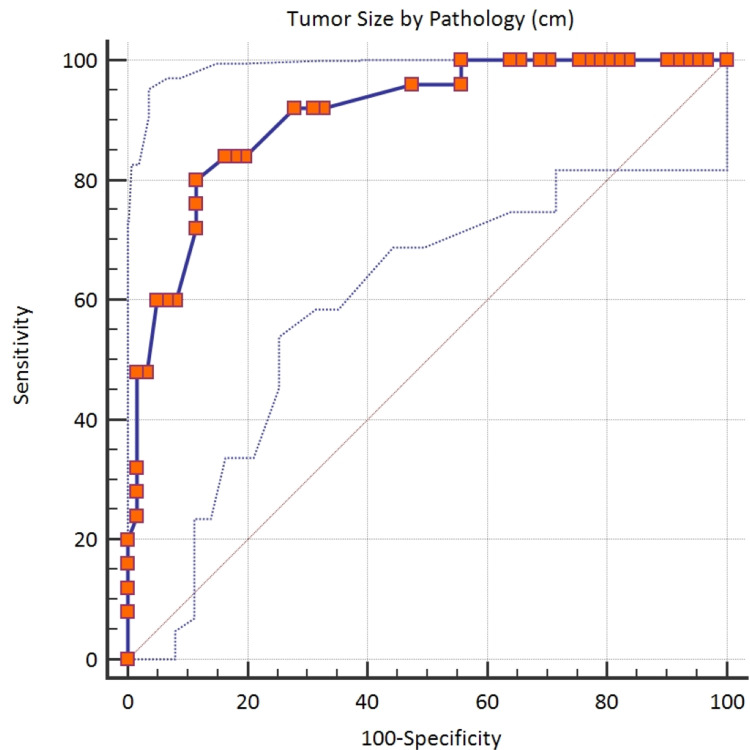
Youden index

According to the Youden index, the cutoff value of >8 cm by histopathology was established as a statistically significant differentiator of lymph node metastasis. The sensitivity and specificity were calculated to be 80% and 88.52%, respectively, with an AUC of 0.907 (Table 5 and Figure 3).

**Table 5:** Youden index

Youden index	0.6852
Associated criterion	>8
Sensitivity	80%
Specificity	88.52%

## Discussion

As the lymphatic system is the most common route of spread in endometrial carcinoma [4], approximately one in 10 patients with endometrial carcinoma who undergo lymphadenectomy have lymph node metastasis. Although lymph node metastasis negatively impacts the survival rate, pelvic lymphadenectomy is associated with various medical and surgical complications [19]. Our study suggests that tumor size (assessed by MRI and histopathology) can be used as a reliable tool to predict possible lymph node metastasis. This may help in incorporating adequate surgical skills and management plans during the treatment of patients with type I endometrial carcinoma.

In our study, of the 25 patients with lymph node metastasis, all had stage IB disease. In 1996, Larson et al. conducted a study on 236 women with endometrial cancer to determine the prognostic significance of myometrial invasion. They also found increased lymph node metastasis (6.4-fold higher prevalence) in patients with >50% myometrial invasion (stage IB) [19].

According to our study, tumor size and lymph node metastasis are positively correlated (r = 0.715, p < 0.01). In their retrospective study in 2005, Shah et al. also indicated that the risk of nodal metastasis increases as tumor size increases and that when the tumor size is larger than 2 cm, the incidence of nodal metastasis is 26.3% [20]. Similarly, in 2014, Cetinkaya et al. also reported that lymph node metastasis is more common in patients with tumors larger than 2 cm [21].

In 2014, Berretta et al. found a higher cutoff value of tumor size of 6.3 cm (±3.1) and decided that a median of 6.5 cm is significantly related to lymph node metastasis. The correlation coefficient was 0.003 (p < 0.01) [4]. We also report a slightly higher cutoff value of >6.5 cm by MRI and >8 cm by histopathology for correlation with lymph node metastasis.

In 2016, Canlorbe et al. studied 633 women with early-stage endometrial carcinoma who were divided into low-risk, intermediate-risk, and high-risk groups. An increased rate of lymph node metastasis was found for a tumor size of >35 mm. This indicates that tumor size is an absolute prognostic factor of lymph node metastasis in women with endometrial carcinoma in the low-risk group [22]. The mean tumor size of patients with lymph node metastasis by MRI and histopathology in our study was 7.86 cm and 10.21 cm, respectively. Therefore, we also found tumor size to be an important predictor of lymph node metastasis. Similarly, in 2015, Mahdi et al. discovered that survival and lymph node metastasis in endometrioid endometrial carcinoma can be predicted by tumor size, considering a tumor size of >5 cm as a predictor of disease-specific survival [15].

Although the FIGO staging system is generally used to stage endometrial carcinoma, it does not include features such as tumor size, tumor location, and peritoneal fluid cytology. In 2013, the European Society for Medical Oncology (ESMO) considered a tumor diameter of >2 cm, myometrial invasion of >50%, histological type, grade 3 disease, and lymph node metastasis as important features of relapse in early-stage disease. Treatment of stage I endometrial cancer includes hysterectomy with bilateral salpingo-oophorectomy with or without lymph node dissection. Lymphadenectomy is significant in assessing prognosis and for future adjuvant therapies [1]. The ESMO-European Society of Gynaecological Oncology (ESGO)-European SocieTy for Radiotherapy and Oncology (ESTRO) consensus conference on endometrial cancer in 2016 also included lymphadenectomy as essential in endometrial carcinoma treatment [23]. Similarly, in 2017, the Spanish Society for Medical Oncology (SEOM) stated that lymphadenectomy is important for evaluating prognosis and considered tumor size of <2 cm, <50% myometrial invasion, and grades I and II as low-risk features of nodal metastasis [24].

We used MRI as an imaging modality since it is the most accurate tool for the preoperative evaluation of patients with endometrial carcinoma [17]. The majority of similar previous studies have either considered that gross pathological specimens correlate with tumor size and lymph node metastasis in patients with type I endometrial carcinoma or they used ultrasound [4,6,15,23]. Similar to the 2017 study, Badawy et al. used ultrasound for the preoperative assessment of tumor size and compared the results with the postoperative histopathological size of the specimens to investigate the correlation with lymphatic spread in patients with type I endometrial cancer. They found both methods to be significantly related to lymph node metastasis with a cutoff value of 4.5 cm by ultrasound and 5 cm by histopathology [6].

Our study showed that in addition to tumor size, grade II (p < 0.01) and stage IB (p < 0.01) tumors were also significantly associated with lymph node metastasis. More recently, in 2021, Oliver-Perez et al. also studied different factors that could be correlated with lymphovascular space invasion and concluded that in type I endometrial carcinoma, involvement of the lower uterine segment and tumor size of >2 cm were independent risk factors for lymphovascular space invasion [25].

The strength of our study was that we used MRI, which is the most appropriate imaging tool for the preoperative assessment of these patients. However, this was a single-center study, which is a limitation. Therefore, further extensive multicenter studies are recommended.

## Conclusions

Our study revealed that tumor size can be successfully used to predict lymph node metastasis in patients with type I endometrial carcinoma. As evident from the results, lymph node metastasis increases with increasing tumor size. MRI is the most accurate imaging modality used to assess tumor size preoperatively. Hence, MRI can be used to incorporate better treatment plans and adequate surgical skills to treat patients with type I endometrial carcinoma.
